# De Novo Sequencing and Hybrid Assembly of the Biofuel Crop *Jatropha curcas* L.: Identification of Quantitative Trait Loci for Geminivirus Resistance

**DOI:** 10.3390/genes10010069

**Published:** 2019-01-21

**Authors:** Nagesh Kancharla, Saakshi Jalali, J. V. Narasimham, Vinod Nair, Vijay Yepuri, Bijal Thakkar, VB Reddy, Boney Kuriakose, Neeta Madan, Arockiasamy S

**Affiliations:** 1 Reliance Technology Group, Reliance Industries Limited, Navi Mumbai 400701, India; Nagesh.Kancharla@ril.com (N.K.); Saakshi.Jalali@ril.com (S.J.); janyavula.v.narasimham@ril.com (J.V.N.); Vinod.Nair@ril.com (V.N.); Vijay.Yepuri@ril.com (V.Y.); thakkar.bjl@gmail.com (B.T.); Neeta.Madan@ril.com (N.M.); 2 AgriGenome Labs Private Limited, Hyderabad 500078, India; vb.reddy@aggenome.com (V.B.R.); boney.k@aggenome.com (B.K.)

**Keywords:** oil seed, biofuel, Euphorbiaceae, genome sequencing, transcriptomics, hybrid sequencing, simple sequence repeats, linkage map, geminivirus

## Abstract

*Jatropha curcas* is an important perennial, drought tolerant plant that has been identified as a potential biodiesel crop. We report here the hybrid de novo genome assembly of *J. curcas* generated using Illumina and PacBio sequencing technologies, and identification of quantitative loci for *Jatropha* Mosaic Virus (JMV) resistance. In this study, we generated scaffolds of 265.7 Mbp in length, which correspond to 84.8% of the gene space, using Benchmarking Universal Single-Copy Orthologs (BUSCO) analysis. Additionally, 96.4% of predicted protein-coding genes were captured in RNA sequencing data, which reconfirms the accuracy of the assembled genome. The genome was utilized to identify 12,103 dinucleotide simple sequence repeat (SSR) markers, which were exploited in genetic diversity analysis to identify genetically distinct lines. A total of 207 polymorphic SSR markers were employed to construct a genetic linkage map for JMV resistance, using an interspecific F_2_ mapping population involving susceptible *J. curcas* and resistant *Jatropha integerrima* as parents. Quantitative trait locus (QTL) analysis led to the identification of three minor QTLs for JMV resistance, and the same has been validated in an alternate F_2_ mapping population. These validated QTLs were utilized in marker-assisted breeding for JMV resistance. Comparative genomics of oil-producing genes across selected oil producing species revealed 27 conserved genes and 2986 orthologous protein clusters in *Jatropha*. This reference genome assembly gives an insight into the understanding of the complex genetic structure of *Jatropha*, and serves as source for the development of agronomically improved virus-resistant and oil-producing lines.

## 1. Introduction

*Jatropha curcas* L. belongs to the Euphorbiaceae family, which has species producing vegetable oils, medicinal, pesticidal industrial chemicals, and various other biologically active compounds [1]. This plant is a shrub which can grow in regions of low to moderate rainfall, and attain a height of 20 feet or more [2]. The shrub is well known for the production of non-edible oil-bearing seeds, and it is preferred for biodiesel production over other food crops. It is now cultivated on a smaller scale in Australia, South Africa, India, Brazil, Fiji, Honduras, Panama, El Salvador, Jamaica, Puerto Rico, and other parts of Caribbean [3]. *J. curcas* can be cultivated on marginal lands and it has other advantages, such as a hardy nature, ease of vegetative propagation, drought tolerance, low seed cost, short gestation period, rapid growth, and easy adaptation to a wide range of agro-climatic conditions [4]. *Jatropha* seed oil contains around 75% unsaturated fatty acids that include oleic, linoleic, and palmitic acids, which makes the oil favorable for bio-diesel production [5,6]. Typical *J. curcas* seeds contain on an average 30–35% oil and 45–50% protein. The fatty acid composition is superior to other vegetable oils in the context of its use as biofuel raw material. Considering the importance of the crop, there is a need for a better understanding of various basic and applied aspects of this plant genome, to improve or develop stable and high-yielding varieties.

Genome assembly is the process of aligning and assembling a large number of DNA fragments that are generated through shotgun sequencing, in order to rebuild the original genome. *Arabidopsis* was the first plant whose genome was sequenced and assembled [7], and the method followed was clone-by-clone sequencing that involved the physical mapping of cosmids or bacterial artificial chromosome (BAC) clones, and further assembly to form cloned contigs. Whole genome shotgun sequencing (WGS) approaches were later adopted to reconstruct the genome, because they obviated the major steps of cloning and physical mapping [8]. The advantage of WGS is that it enables the examination of entire genomic content of an organism at once, without the need to depend on assembled cloned contigs. A vast number of plant genomes have been successfully sequenced using WGS [9]. De novo genome assembly is used to assemble the genome of a novel organism, which is not similar to that of any existing organism. This process involves assembling single reads into contiguous sequences (also known as contigs), which are then extended in the 3′ and 5′ directions by overlapping other sequences [10]. Cucumber [11] and apple [12] plant genomes have been sequenced using the combination of pyrosequencing and Sanger sequencing.

The whole genome sequencing of *J. curcas* using next-generation sequencing (NGS) technology such as Illumina was reported by Hirakawa and Wu groups in 2012 and 2015, respectively [2,13,14]. Further, these reports were mainly based on single tissue-specific samples, and they were observed to have high numbers of contigs, low N50 values and a smaller number of protein coding genes. However, to gain an in-depth knowledge of *J. curcas*, one needs to use the latest sequencing technologies to cover the maximum genome size, to further validate the differential expression of protein coding genes for traits or conditions of interest.

Due to the rapid development of NGS technologies, hybrid-based methods, such as sequencing and assembly based on the combination of long and short reads are presently adopted [15]. The hybrid approach, which is used to assemble genomes, involves supplementing short, accurate second-generation sequencing data with long, relatively less accurate third-generation sequencing data to resolve complex repeated DNA segments [16]. The main limitation of single-molecule third-generation sequencing that prevents it from being used alone is its relatively low accuracy, which causes inherent errors in the sequenced DNA. Assembling a genome by using only second-generation sequencing techniques can lead to the exclusion of important aspects of the genome, thus resulting in an incomplete assembly. Hence, the supplementation of third-generation reads with short, high-accuracy second-generation sequences can eliminate these inherent errors, and help in obtaining crucial details of the genome [17]. We have thus adopted a hybrid assembly method of genome sequencing to capture and capitalize on the accuracy of the second-generation method of Illumina sequencing, and the long read capability of third generation method of PacBio (Pacific Biosciences, Menlo Park, CA, USA) sequencing. This was expected to result in more accurate forecasts of the genome. This can be strengthened and verified by the transcriptome data of differentially active tissues from the seed stage to the mature plant, covering all developmental stages of the plant (current study).

Genomic selections improve breeding strategies based on genomic information coupled with estimated breeding values assigned to markers to increase the rate of genetic gain [18]. Such applications have been utilized in genetically improving the crop plants, e.g., in maize and rice [19].

Here, we report the *J. curcas* genome assembly using hybrid sequencing methodology, which revealed 84.8% gene space completeness by using Benchmarking Universal Single-Copy Orthologs (BUSCO) analysis [20,21,22] in addition to the identification of quantitative trait loci for *Jatropha* Mosaic Virus (JMV) resistance. Firstly, we generated WGS, paired-end (PE), and mate pair (MP) sequence reads (Illumina platform), as well as long-base paired reads by Pacific Biosciences from the genomic DNA, and then we built a hybrid de novo assembly. Further, we constructed an RNA sequencing (RNA-seq) library of 13 different tissues (at various growth stages of plant) obtained from Illumina short-read sequences of complementary DNA (cDNA) from *J. curcas*, to validate the predicted genes from hybrid genome assembly. Thereafter, we identified simple sequence repeats (SSR) and designed SSR markers to analyze the genetic diversity, and to generate linkage maps and quantitative trait locus (QTLs) for geminivirus resistance. In addition, we examined for conserved genomic information between *J. curcas* and the publicly available genomes of 13 related and relevant plant species (listed in the Methods, Section 2.12). The genome analyses presented in this study provides a rich resource of genetic information for advanced *J. curcas* breeding and genetic improvement programs. Our study is the first one to report QTLs for geminivirus resistance from a de novo hybrid assembly of the *Jatropha* genome.

## 2. Materials and Methods 

### 2.1. Plant Materials

We selected the inbred line RJC1 of the *J. curcas* developed at the Agricultural research farm in Andhra Pradesh for our whole-genome and transcriptome sequencing. RJC1 was chosen for WGS because of its high general combing ability (GCA) and homozygous genome. The underlying assumption is that lines with high GCA tend to possess useful and well conserved regions which are of great value in developing lines and hybrids in plant breeding. Healthy and disease-free leaf samples were collected for DNA isolation and 13 different plant tissues of RJC1 at various developmental stages were collected for RNA isolation (as listed in Table S1). The DNA and RNA extractions were carried out by AgriGenome Labs Pvt. Ltd. (AGL, Hyderabad, Telangana, India) by following their in-house protocol. Geminivirus resistance assay for the two parental lines, namely *Jatropha integerrima*, *J. curcas*, and its interspecific F_2_ mapping populations was determined by PCR and cleft grafting methods. In the cleft grafting method, a highly geminivirus-susceptible *Jatropha* genotype was used as the root stock and scions of test parental lines, and the progenies were grafted into three biological replicates for geminivirus phenotyping [23]. The phenotypic symptoms of JMV were scored after 45–60 days of grafting to identify resistance or susceptibility of the parents and progenies tested. Disease instance and symptom severity were recorded according to the disease severity scale. Universal degenerate primers of geminivirus, targeting the coat protein gene, were utilized in screening the parental lines and grafted plants [24].

### 2.2. DNA Sequence Data Generation

The total genomic DNA was isolated, and 5 µg high-quality genomic DNA was used for library preparation by AGL. Fragment libraries were prepared using Illumina TruSeq (San Diego, California, United States) for all samples and validated on the Agilent (Santa Clara, CA, United States) Technologies 2100 Bioanalyzer using the Agilent DNA chip. The short read was done at a coverage of 100× while the PacBio RS long read was done at a coverage of 8× Three PE Illumina-based DNA libraries with an insert size of 150, 300, and 500 bp were generated. Illumina MP libraries were also prepared with an average insert size of 1.5 kb using the Nextera (San Diego, CA, USA) mate-pair sample preparation kit by following the manufacturer’s protocols [25]. Sequencing of the whole genome was carried out using Illumina HiSeq 2500 technology for PE, and MiSeq for MP to build a draft genome. The *J. curcas* whole genome was also sequenced using eight SMRT (Single Molecule Real-Time) Cells using the MagBead (Menlo Park, CA, USA) OneCellPerWell protocol with a 20 kb library size and P6-C4 chemistry on the PacBio RS II platform (Pacific Biosciences).

### 2.3. Sequence Data Trimming, Filtering, Error Correction, and Assembly

Quality checking and trimming of Illumina reads were performed by FASTQC [26] and CutAdapt [27]. All the low-quality data was filtered out using sickle v.1.33, and all duplicate reads and reads of length less than 30 bp were discarded using FastUniq to avoid error in scaffolding for the whole genome sequencing and genomic variations [28]. Genome estimation was performed by Kmer optimization using Kmer-genie [29]. High-quality unprocessed Illumina reads were assembled with Maryland Super-Read Celera Assembler (MaSurCA) by using the default parameters [30].

Initially, we made a genome assembly with Illumina short-read sequences using well-known assemblers like the Sparse assembler [31,32], SOAPdenovo2 [33], and MaSuRCA. The long reads generated by PacBio sequencing were utilized to fill the gaps in Illumina-based assembly. Since the long PacBio reads contain high error rate, it was essential to correct those long reads by using a PacBioToCA pipeline, which is a part of the Celera assembler (version 8.3). We integrated all three Illumina-based assemblies with PacBio data individually, to complete the respective hybrid genome assemblies by using default parameters of the DBG2OLC algorithm, in order to enhance the short-read assemblies. DBG2OLC is an efficient assembler for attaining high quality assemblies in a shorter time period with long reads [34]. Further, the individual hybrid genome assemblies were improved by RNA scaffolding of long transcriptome data, using L_RNA_scaffolder [35]. We further selected the genome assembly with the highest N50 value and the lowest number of contigs for downstream analysis. The BUSCO pipeline version 3 (released July 2017) and Core Eukaryotic Genes Mapping Approach (CEGMA) were finally used to assess the gene space completion of the selected hybrid genome assembly [36,37].

### 2.4. Gene Prediction 

We predicated coding sequences (CDS) from the assembled contigs using the AUGUSTUS (3.2.1) pipeline [38], and BRAKER2 (v2.1.0) [39]. We used the AUGUSTUS pipeline for ab initio gene prediction based on default parameters, and by including *Arabidopsis* plant genes. We considered genes for further downstream analysis, which were predicted with the AUGUSTUS pipeline. The BRAKER2 pipeline was used to predict de novo genes, using RNA-seq as hits for unsupervised genome annotation. We also predicated transfer RNA (tRNA) genes from the assembled contigs, using the tRNAscan-SE program [40].

### 2.5. Gene Annotation

The predicted CDS from the assembled contigs were used for annotation with CANoPI (Contig annotator pipeline, AGL in-house pipeline). The predicted CDS were then compared with the National Center for Biotechnology Information non-redundant (NCBI nr) [41] database, using the BLASTx program with an Ee-value ≤ 1e^−5^. The Gene Ontology (GO) terms for the predicted CDS were extracted on the basis of *Arabidopsis* GO data. We annotated all the genes in terms of GO terms such as molecular function (MF), cellular component (CC), and biological process (BP).

### 2.6. Transcriptome Sequencing and Data Generation

The total RNA was extracted from 13 different plant tissues of RJC1, and verified on an Agilent Technologies 2100 Bioanalyzer using the Agilent RNA chip manufactured by AGL. For our study, total RNA with an RNA Integrity Number (RIN) greater than or equal to 8 (high quality) was considered for library preparation. An Illumina-based library was constructed using TruSeq RNA Sample Prep Kits, as per the recommendations of the manufacturer [Illumina: Catalog # RS-930-2001]. The constructed libraries were sequenced on an Illumina HiSeq 2500 to obtain 2 × 100 bp PE reads per sample. The sequencing reads were quality-checked, based on base quality, base compositions, and GC distribution. 

### 2.7. Ab Initio Gene Model Validation

We used RNA-seq data to validate genes that were predicted by the AUGUSTUS pipeline from assembled contigs. High-quality reads of RNA-seq of RJC1 from different development stages, ranging from germinating seed to vegetative and flowering stages, to the geminivirus-affected tissues data were aligned with genome sequence using Bowtie2 software [42]. TopHat2 in combination to Bowtie2 aligner was used to assemble the RNA-seq reads onto the genome assembly to identify splice junctions [43]. We also calculated the coverage of each gene using the Bedtools software [44]. Differential expression analysis between the geminivirus-infected and non-infected leaves was performed using the cuffdiff program of the cufflink package, with default parameters. The differentially expressing genes (DEGs) were further mapped to Kyoto Encyclopedia of Genes and Genomes (KEGG) pathways using *Arabidopsis* as model organism.

### 2.8. Identification of Simple Sequence Repeats

We used hybrid genome assembled contig sequences to identify microsatellite sequences with MISA (MIcroSAtellite identification) tool by applying default parameters [45]. Primer pairs for amplification of the SSR-containing regions were designed on the basis of the 250 bp flanking sequences of each primer with the batch primer program.

### 2.9. Genetic Diversity Analysis

A total of 49 *Jatropha* accessions were utilized in this study; which included *J.curcas*, *J. integerrima* and *Jatropha gaumeri* species obtained from various regions such as Asia and Central America. These were established at our agriculture research farm in Samalkota, Andhra Pradesh, India, and leaf tissues were collected for this study. The region-wise list of accessions used are given in Table S2. The genetic characterization of *Jatropha* elites was analyzed with 120 SSR markers, designed from the assembled genome sequence of RJC1. The allelic data obtained from these polymorphic markers were computed to generate a dendrogram using unweighted pair group method with arithmetic mean (UPGMA) cluster analysis of NTSYS pc 2.02 software [46]. 

### 2.10. Linkage Mapping and Quantitative Trait Locus Analysis for Jatropha Mosaic Virus Resistance

We generated 282 F_2_ progenies from an interspecific cross involving *J. curcas* and *J. integerrima* as parents. We utilized 2784 SSR markers designed from WGS assembly for the identification of polymorphic markers between parental lines. The identified polymorphic markers were further utilized in the genotyping of 282 F_2_ progenies. This genotypic data was further utilized in generating linkage map using JoinMap 4.1 software [47]. The genotype data were correlated with phenotypic data, and QTL analysis was conducted using Cartographer V 2.5 [48] to identify the genetic factors controlling JMV resistance in *Jatropha*.

### 2.11. Identification and Annotation of Repetitive Sequences

We used a de novo approach to identify repeat elements within the *J. curcas* genome, using the RepeatScout software package [49]. This program was run using the default parameters, to build an initial frequency table that was specific to our genome. In RepeatScout analysis, it further utilizes the frequency table and produces a fasta file containing all the repeats, which are then filtered out to remove low-complexity and tandem repeats. Finally the program filters the generated library to remove repeat elements that does not fulfill criteria (default = 10) [50].

### 2.12. Comparative Genomic Analysis

*Jatropha curcas* gene sequences predicted from the AUGUSTUS (3.2.1) pipeline were translated into amino acid sequences, and comparative genomic analysis for selected metabolic pathways was performed using the KEGG [51] database. On the basis of the BlastKOALA pipeline [52] we distributed amino acid sequences into different metabolic pathways. *Jatropha* being a biofuel crop, our key interest in comparative genomic studies was to analyze lipid-metabolizing genes across different plant species. We considered three categories of plants for this study which includes oil-producing plants (*Brassica napus*, *Brassica rapa*, *Arachis hypogaea*, *Helianthus annuus*, and *Ricinus communis*), oil-producing algae (*Botryococcus braunii*, *Chlamydomonas reinhardtii*, *Monoraphidium neglectum*, and *Nannochloropsis gaditana*) and model plant species (*Oryza sativa*, *Zea mays*, *Medicago truncatula*, and *Arabidopsis thaliana*). Sequences of these 13-plant species were downloaded from the public databases, BLAST analyses were performed, and results were filtered at a sequence similarity of 80%.

To understand the functional relatedness based on sequence conservation, we performed orthologous protein clustering with five different plant species, including *Glycine max*, *Arabidopsis thaliana*, *Arachis hypogaea*, *Brassica rapa*, and *Chlamydomonas reinhardtii* with *J*. *curcas*, using OrthoVenn [53]. We were also interested in comparing *Jatropha* at the complete protein level with other oil-producing species, such as *Ricinus communis*, *Brassica napus*, *Brassica rapa*, *Helianthus annuus*, *Arachis hypogaea*, and *Arabidopsis thaliana*, to identify conserved proteins across these plant species. Sequences having 100% alignment with *Jatropha* were considered for functional conservation analysis.

### 2.13. Data Availability

The whole genome sequence assembly data generated during the current study is available as an archive in NCBI-Genome: BioProject ID: PRJNA470761 and BioSample ID: SAMN09197287.

## 3. Results

### 3.1. Whole Genome Sequencing Statistics

In our study, 58.3 Gbp high quality genome sequence data from one MP and three PE Illumina libraries was generated. The GC content of the raw data obtained from Illumina ranged from 36 to 38%. In addition, 5.2 Gbp of long reads were also generated through the PacBio RS II platform, which were good quality data with an average read length of >10 kb and a GC content of 36% (Figure S1). The statistics of the raw reads for PE, MP, and PacBio are presented in Tables S3.1 and 3.2.

### 3.2. Sequence Data Trimming, Filtering, Error Correction, and Assembly

We captured a total of 220,870,105 high-quality reads from three different PE libraries and 8,301,438 MP reads. A total, 726,977 PacBio reads were generated (Table S4), which were further corrected using the pacBioToCA pipeline, and 1.85 Gbp of error-free sequences were extracted. Table S5.1 gives the statistics of high-quality Illumina clean error-free reads and Table S5.2 details PacBio sequences. From the filtered Illumina reads, we estimated the genome size to be 265 Mbp, with the help of an 82 kmer value.

**Figure 1:**
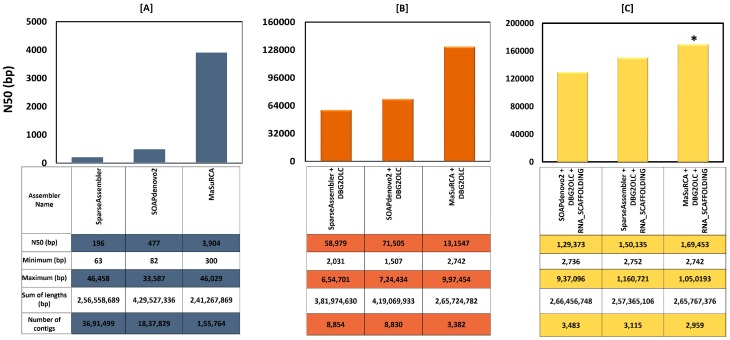
Statistics of the assemblers used in our study for (**A**) single assembly (in blue), (**B**) hybrid assembly (in orange), and (**C**) hybrid assembly with RNA scaffolding (in yellow). The table below the bar chart shows the statistics for each of the assembler(s). The combination of assemblers marked with a star (*) was used for our analysis.

We observed better genome assembly statistics with MaSurCa (N50:3,904; contig # 1,55,764) when compared to SOAPdenovo2 (N50:477; contig # 1,837,829) and SpareseAssembler (N50:196; contig # 3,691,499). Using MaSurCa software, we observed the best Illumina-based assembly of *J. curcas*, having a coverage of 241.2 Mbp genome length and 32.49% GC content. (Figure 1A) The hybrid genome assembly statistics of MaSurCA + DBG2OLC (N50:1,31,547; contig # 3,382) was observed to be superior to the other two assembly combinations (SOAPdenovo2 + DBG2OLC (N50:71,505; contig # 8,830) and SpareseAssembler + DBG2OLC (N50:58,979; contig # 8,854)) assessed (Figure 1B). Further, the final hybrid genome assembly with MaSurCA + DBG2OLC+RNA scaffolding resulted in the enhancement of the assembly statistics (N50:1,69,453; contig # 2,959) compared to other two assemblies (Figure 1C) (Table 1).

The statistics of the assemblers used in our study for both single and hybrid assemblies are shown in Figure 1. In the BUSCO and CEGMA analysis, we used 429 and 248 ultra-conserved core eukaryotic genes respectively, and then mapped them against the hybrid assembly. CEGMA analysis showed that 84.2% of core eukaryotic gene sequences were complete (94.76% were partial), and BUSCO analysis revealed that 84.8% of eukaryotes gene sequences were complete in our assembly. This BUSCO analysis revealed that the lengths of the restored genes are found to be within two standard deviations of the BUSCO group mean length to be considered as complete and passed the minimum cut-off score. This depicts that our genome assembly is relatively complete with respect to the gene space [33]. The same pattern has been observed in *Hevea brasiliensis* genome assembly wherein 79% complete gene space observed in BUSCO analysis [21,36]. The percent of gene space observed in our study is relatively high, but slightly lesser than *Ipomoea nil*, where 95% gene space is reported [54]. The difference could be due to various factors, such as complexity of the genome and/or repetitive elements. 

### 3.3. Prediction of Genes from Assembled Genome

We predicted a total 20,759 genes from the hybrid-assembled genome against the default *Arabidopsis* genes, using the AUGUSTUS pipeline with a total length of 40.7 Mbp. The identified CDS have an average length of 1.1 kb and 43% GC content. For de novo gene prediction, we utilized the 37.66 Gbp of high-quality RNA sequences obtained from 13 different growth stage tissues of *J. curcas* to generate complete gene structures for the assembled *Jatropha* genome, using BRAKER2 pipeline along with GeneMark-ET and AUGUSTUS (3.2.1) packages. With BRAKER2 analysis, we predicted a total of ab initio 18,890 genes using RNA-seq unsupervised genome annotation. We masked the repeat regions using RepeatScout before predicting genes from assembled *Jatropha* genome.

### 3.4. Gene Annotation and Kyoto Encyclopedia of Genes and Genomes Pathway Analysis

We predicted 20,759 CDS from the AUGUSTUS pipeline, and compared them with the NCBI nr database using BLASTx program, and found at least one hit for 17,703 of the predicted CDS. However, at the protein level, 85.28% of the CDS showed more than 60% similarity with the existing proteins of the NCBI database. While, 13,078 CDS sequences were annotated using the UniProt Database [55,56], amounting to 63% of annotated *Jatropha* proteins. From the assembled contigs, we further extracted 658 tRNA genes by using tRNAscan-SE.

**Figure 2:**
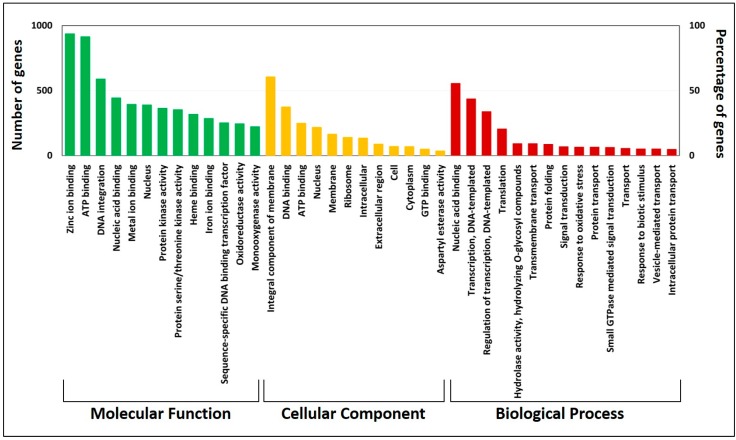
The bar chart depicts the top 15 Gene Ontology (GO) annotation categories, including molecular function (in green color), cellular component (in yellow color), and biological function (in red color).

From the BLASTx results, we observed that out of the top 15 hits, 86% of genes belongs to the *Jatropha* species followed by *Arabidopsis*. We also extracted possible GO terms for the predicted CDS. The total number of GO terms identified in molecular function (55.94%), biological process (22.39%), cellular component (21.66%), and the top 15 terms of the GO categories are shown in Figure 2. We observed that molecular function had the majority of genes (5726) distributed across functions such as ATP binding (15.99%) and zinc binding (16.3%). We found zinc binding- and ATP binding-related genes to be more commonly expressed as molecular function in our analysis, possibly due to the phytoremediation and drought tolerance of *Jatropha*, as reported previously [56,57]. Further characterization of individual genes is required to confirm the functional role in predicted GO category.

We extracted amino acid sequences from AUGUSTUS-predicted CDS sequences and categorized them in the KEGG database. Out of 20,759 CDS sequences, 6,815 amino acid sequences were annotated and distributed into 15 KEGG categories. Majority of amino acid sequences fell in category global and overview maps, followed by the other categories mentioned below in a descending order of the number of annotated amino acid sequences: genetic information processing, cellular processes, environmental information processing, carbohydrate metabolism, amino acid metabolism, energy metabolism, lipid metabolism, biosynthesis of other secondary metabolites, metabolism of cofactors and vitamins, metabolism of other amino acids, metabolism of terpenoids and polyketides, nucleotide metabolism, glycan biosynthesis and metabolism, xenobiotics biodegradation, and metabolism. The total number of KEGG terms identified in the 15 different categories is shown in Figure S2.

### 3.5. Transcriptome Sequencing and Gene Validation

We performed transcriptome sequencing for 13 different *Jatropha* tissues using an Illumina Hiseq2500 to generate 2 × 100 short reads. The aim of RNA-sequencing was to validate the gene models that were predicted from the whole genome sequencing. Further, our aim was to observe the differential gene expression between geminivirus-infected and healthy leaves of RJC1. We generated a total of 49 Gbp data using RNA-sequencing, and the statistics of the corresponding 13 different *Jatropha* tissues from development stages, ranging from germinating seeds to vegetative and flowering stages, to the geminivirus-affected tissue, as given in Table S1. Genes were predicted using the AUGUSTUS software for the RNA-seq data. By aligning RNA-seq data to ab initio genes, we were able to capture 96.46% of *Jatropha* genes with a significant depth and coverage of >10%; while only 4% of the ab initio genes could be validated. Gene coverage for individual genes was calculated using bedtools, and it was observed that 89% of genes had a coverage of more than 50%. The sequence alignment percentage for geminivirus-infected and healthy (non-infected) leaves with RJC1′s WGS was 54.84 and 60.6% respectively. We performed differential gene expression analysis between the geminivirus-infected and non-infected leaves of *J. curcas*, using the cuffdiff program of the cufflink package with parameters of *p*-value < 0.05, False Discovery Rate (FDR) < 0.05, and Fragments Per Kilobase of transcript per Million mapped reads (FPKM) > 0, which resulted in the identification of 379 upregulated (log2 fold-change ≥ 1) and 241 down regulated (log2 fold change ≤−1 ) genes (Figure S3).

From KEGG pathway analysis, out of 620 DEGs, only 12 DEGs (two down-regulating and 10 up-regulating, Table S6) specifically fell into pathways such as thiamine metabolism [57], terpenoid backbone biosynthesis [58], MAPK signaling pathway [59], oxidative phosphorylation [60], carbon fixation in photosynthetic organisms [61], and plant hormone signal transduction. These genes are mainly found to be involved in biotic stress-related pathways, for example, terpenoid backbone biosynthesis, which generally produces terpenes compounds to combat the attack of herbivorous insects on plants [58].

### 3.6. Characterization of Genes Specific to the Drought Condition

*Jatropha* is known for drought tolerance, we cross-checked the *Jatropha* genome sequence with publically available plant drought-responsible genes, which are listed in droughtDB [62]. We identified a total of 3,535 genes of *Jatropha* that have homology based on sequence alignment. On the basis of characterization specified by the database, we classified the number of genes that are associated with physiological adaptation and molecular adaptation. Out of the 3,535 genes, 667 were categorized into ion and osmotic homeostasis, 28 into growth control, 196 into detoxification, 431 into functional proteins, 632 into gene expression, 107 into post-translation modification, 1,156 into signal transduction, 282 into hormone signaling, and 36 into acid anhydride hydrolases. These genes with their characterizations are listed in Table S7.

### 3.7. Microsatellite Primer Designing 

Microsatellites were identified and localized from the assembled contigs by using MISA software. We classified the identified marker sequences on the basis of the nucleotide repeats. From identified marker sequences, we designed primer sequences using batch primer software [63]. We observed a greater number of di-nucleotide (18,300) repeats, followed by tri- (8,574), tetra- (1,557), penta- (257), and hexa- (122) repeats. Table S8 gives the statistics of marker sequences, and the designed primer-pairs for different types of SSR repeats. Out of the 18,300 di-nucleotide microsatellite markers predicted, we designed 12,103 SSR markers. We shortlisted 2,784 of them on the basis of their proximity to nearby genes by up to 1 kb for downstream analysis.

### 3.8. Genetic Diversity Analysis

The PIC (polymorphic information content) was calculated for 120 SSR markers, and RJM54 found to exhibit the maximum PIC value of 0.49. UPGMA cluster analyses resulted in two major groups, which included 37 *J. curcas* elites, eight *J. integerrima* elites, and four *J. gaumeri* elites. Jaccard’s similarity coefficient ranged from 0.22 to 0.92, with a mean genetic similarity of 0.57 at all SSR loci. A dendrogram for 49 *Jatropha* lines derived from UPGMA cluster analysis is shown in Figure S4. Through this analysis, we were successfully able to reduce the crossing load by 40–60% by eliminating crosses from a closer genetic background. 

### 3.9. Linkage Mapping and Quantitative Trait Loci Analysis

**Figure 3:**
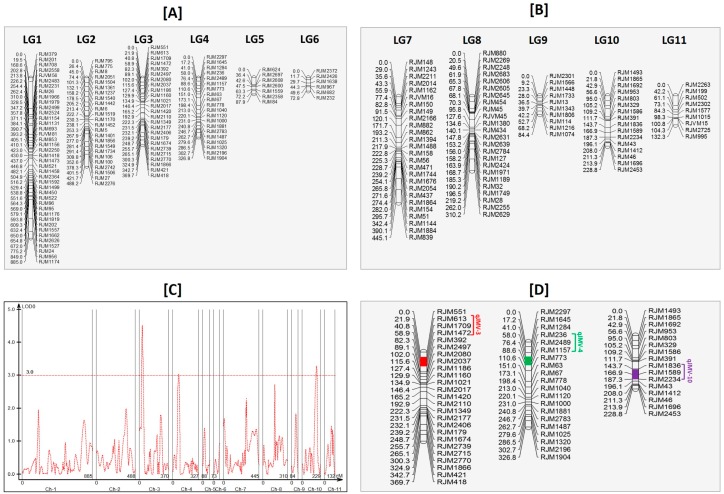
An interspecific genetic linkage map (**A**) LG1–LG6; (**B**) LG7–LG11 of *Jatropha curcas* based on an F_2_ mapping population generated by crossing *J.curcas* × *J. integerrima*. Estimates of map distances between markers are indicated in Kosambi centimorgans; (**C**) Whole genome scan of quantitative trait loci (QTLs) for *Jatropha* Mosaic Virus (JMV) resistance in *Jatropha* linkage maps. The X axis represents the distance in centimorgans (cM), and the Y axis depicts the logarithm of the odds (LOD) score. The red horizontal dotted line represents a significance LOD threshold value of 3.0; (**D**) Summary of three minor QTL locations detected in the genome of *Jatropha*. QTLs, represented by bars, are shown on the left side of the linkage groups.

In parental polymorphic studies, we found 238 SSRs are polymorphic between the parents (~8%) out of 2784 markers screened. Thirty-one polymorphic markers could not be mapped onto linkage maps, due to segregation distortion, and the remaining 207 SSR markers were mapped onto 11 linkage groups (Figure 3A,B). The length of 11 linkage maps ranged from 72.8 to 885 cM. The linkage map covered 3431.2 cM with an average marker spacing of 16.5 cM ranging from 8.4 to 22.1 cM. The highest density of 40 markers was observed on linkage group 1, and the lowest density of six markers was observed on linkage group 6 (Table S9). A logarithm of the odds (LOD) cutoff of 3.0 was taken for QTL identification (Figure 3C). The analysis revealed three minor QTLs in linkage groups 3, 4, and 10, with LOD values of 4.5, 3.1, and 3.3, respectively (Figure 3D). Being minor QTLs, these were named qJMV3.0, qJMV4.0, and qJMV10, with phenotypic variances of 8, 9, and 9, respectively. We also observed some minor QTLs ranging from 2.2 to 2.9 LODs in linkage groups 7 and 8. The marker intervals for the three QTLs ranged from 30.6 to 43.6 cM. The marker intervals and putative genes available in these QTL regions are listed in Table S10. 

### 3.10. Repetitive Sequences in Whole Genome Sequencing

The *Jatropha* genome contains a large number of transposable elements, but only a small number of them have been reported in the public domain [14]. We therefore, used a de novo approach to construct a repeat library of 631,842 elements, including interspersed and simple repeats. In total, 109.2 Mbp of repeat elements were identified, indicating ~41.2% of the genome to be repetitive. 

### 3.11. Comparative Analysis of Lipid Metabolism Genes

**Figure 4:**
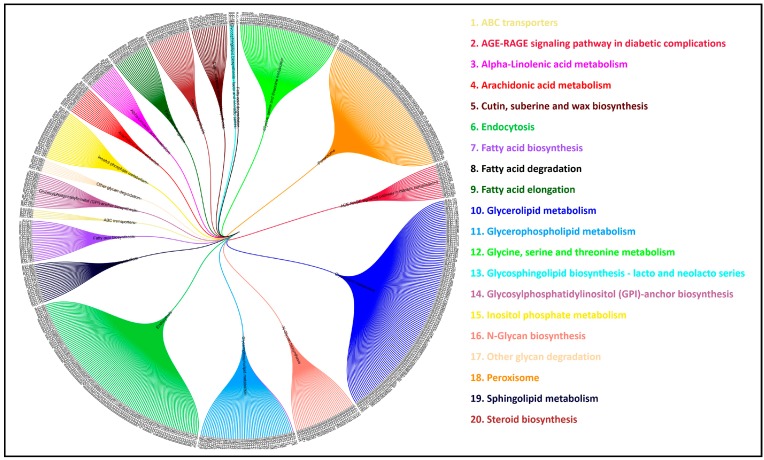
The 20 lipid metabolizing pathways and their components (as labelled 1–20), with the outer circle representing the individual RJCL genes that are involved in respective activities. In total, 693 lipid-metabolizing genes of RJC1 (RJCL: *Jatropha* Cultivar1 Lipid; RJCL1-RJCL693) are shown in outer-most circle.

**Figure 5:**
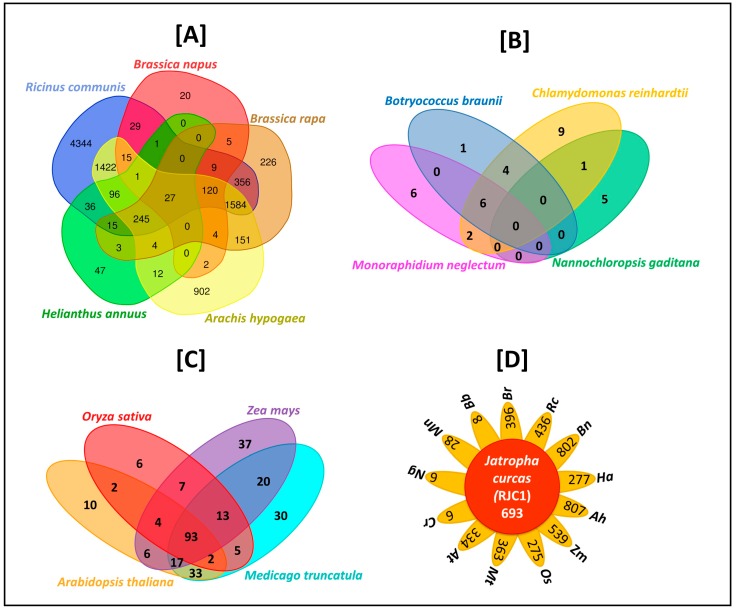
Venn diagrams depicting the comparative analysis for 693 conserved lipid-metabolizing genes of *J.curcas* between (**A**) Oil-producing plants (*Brassica napus*: *Bn; Brassica rapa: Br*; *Arachis hypogaea*: *Ah*; *Helianthus annuus*: *Ha*, and *Ricinus communis*: *Rc*); (**B**) Oil-producing algae (*Botryococcus braunii*: *Bb*; *Chlamydomonas reinhardtii*: *Cr*; *Monoraphidium neglectum*: *Mn*, and *Nannochloropsis gaditana*: Ng); (**C**) Model plant species (*Oryza sativa*: *Os*; *Zea mays*: *Zm*; *Medicago truncatula*: *Mt*, and *Arabidopsis thaliana*: *At*). Plot; (**D**) shows the 13 plant species having gene conservation with 693 RJC1 lipid-metabolizing genes. The middle circle depicts a total of 693 RJC1 lipid-metabolizing genes, and leaves depicts the individual gene numbers of each of the 13 plant species.

Through KEGG pathway analysis, we identified 693 genes that are specifically involved in 20 different lipid metabolic pathways and their corresponding components (Figure 4). We applied stringent conditions to compare the sequences, and identified 27 conserved gene sequences across all the oil producing species selected in this study. The majority of the genes (1584) were conserved across three species, *Ricinus communis*, *Brassica rapa*, and *Arachis hypogaea*, and the highest number of conserved genes of *Jatropha*, 4344, was shared with *Ricinus communis* (Figure 5A–D), whereas *Jatropha* showed conservation of only a few genes with algal species (Figure 5B). Moreover, comparative genomics study showed that 93 genes of *Jatropha* are conserved among model plant species such as *Oryza sativa*, *Arabidopsis thaliana*, *Zea mays*, and *Medicago truncatula*, and Figure 5D shows the number of conserved genes of 13 different plant species that are involved in the lipid metabolism of *J. curcas*.

**Figure 6:**
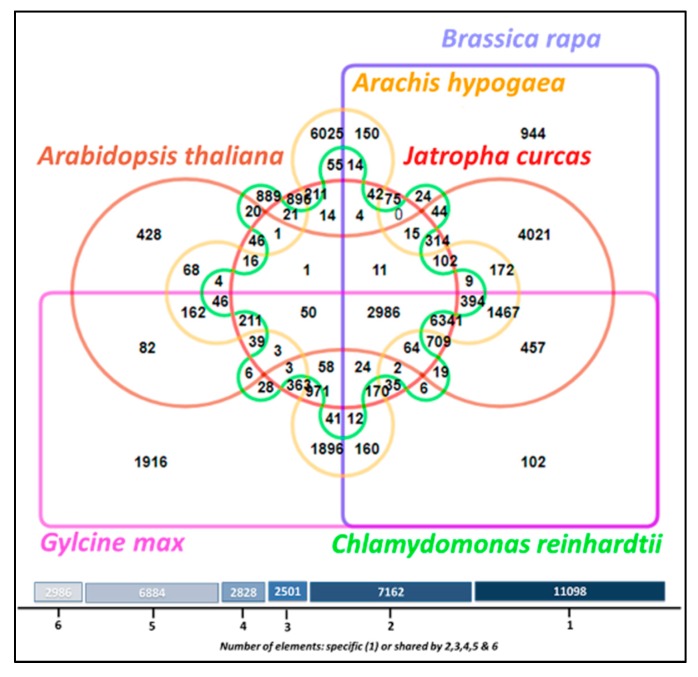
Venn diagram representing the orthologous protein clusters shared among *J. curcas*, *Glycine max*, *Arabidopsis thaliana*, *Arachis hypogaea*, *Brassica rapa*, and *Chlamydomonas reinhardtii*.

**Figure 7:**
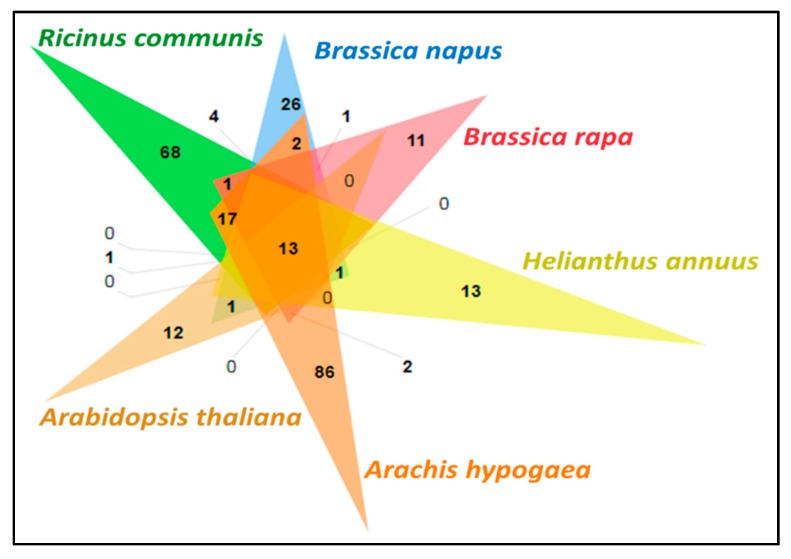
Comprehensive comparative protein analysis of RJC1 with *Ricinus communis*, *Brassica napus*, *Brassica rapa*, *Helianthus annuus*, *Arachis hypogaea*, and *Arabidopsis thaliana*.

After comparing the whole genomes of *Glycine max*, *Arabidopsis thaliana*, *Arachis hypogaea*, *Brassica rapa*, and *Chlamydomonas reinhardtii* with that of *J. curcas*, we identified 2986 orthologous protein clusters shared among five species. *Jatropha’s* maximum protein clustering was observed with *Glycine max* (363 proteins), and its minimum protein clustering with *Chlamydomonas reinhardtii* (21 proteins) (Figure 6). Further, we performed comprehensive comparative protein analysis, and observed 13 proteins of *Jatropha* that were shared across *Ricinus communis*, *Brassica napus*, *Brassica rapa*, *Helianthus annuus*, *Arachis hypogaea*, and *Arabidopsis thaliana* (Figure 7). The majority of the observed functions included polyubiquitin protein types that are mainly interfering cell signaling systems of plants, aiding them in transforming metabolic pathways for defending against abiotic stresses.

## 4. Discussion

This report presents the genome of the biofuel crop *Jatropha*, and the application of markers for the development of QTLs for geminivirus resistance. The availability of comprehensive genome sequences in the public domain opens new avenues for its genetic improvement by the world scientific community. To date, approximately 249 plant species have been sequenced using various sequencing technologies, as stated in the latest reports of NCBI-NIH. Among these, the sequencing of commercially important plant species such as rice (*Oryza sativa*), cotton (*Gossypium hirsutum*), neem (*Azadirachta indica*), wheat (*Triticum aestivum*), rape (*Brassica napus*), papaya (*Carica papaya*), corn (*Zea mays*), and castor (*Ricinus communis*) has gained much importance because of their positive economic value in the market. *Jatropha* is a potential biofuel crop with unlimited economic potential, as it has fuel value. A new paradigm is needed to address or overcome the current hindrances in developing high-yielding, disease-resistant *Jatropha* hybrids. *Jatropha* is severely affected by *Jatropha* mosaic disease, collar rot (Fungal disease), and insect pests like mealy bugs, scales etc. *Jatropha* mosaic disease is caused by geminivirus, and collar rot is caused by a consortium of fungi; both have the potential to reduce yields to an extent of 80% and 50%, respectively, based on the field data recorded at our research and development farms. There is still a big gap in understanding the complete potential of *J. curcas* because of several technological and economic reasons, in addition to the limited knowledge that is gathered on the genetics and crop husbandry of *Jatropha* [64]. There is an immense and immediate need for understanding the *Jatropha* plant at the genome level to upgrade the plant in terms of more number of female flowers per inflorescence, more number of inflorescences per branch, more numbers of productive branches, continuous flowering, high seed yield with high oil content, early maturity, and resistance to pests and diseases, drought and cold tolerance, more efficient canopies with best possible source-sink ratios, etc. The hybrid genome sequence of a *Jatropha* species constitutes an important resource to study the genome evolution, not only in Euphorbiaceae, but also in other closely-related families. The first gene discovery of *J. curcas* was performed by sequencing ESTs from a full-length cDNA library of developing seeds; however, only 7,009 unigene coding genes could be identified [65].

In our study, we have applied a novel hybrid sequencing approach, wherein short and long reads were assembled simultaneously to improve the quality of the sequenced genome of *J. curcas* elite RJC1 resulted in the achievement of 84.8% of the *J. curcas* gene space, comprising 20,759 annotated genes, which was assembled through 2959 contigs. Also, 96.46% of the predicted genes were further re-confirmed by aligning them to the RJC1 RNA-seq data from 13 different tissues. The assemblers used for the de novo assembly of Illumina reads include Sparse assembler, SOAPdenovo2, and MaSuRCA. The assembly statistics for MaSuRCA was observed to be highest as shown in Figure 1A with respect to N50 value and the number of contigs. The N50 value achieved using MaSuRCA was found to be 10–15 times higher than the value given by Sparse and SOAPdenovo2 assembler. This was made possible by increased efficiency of the de Bruijn graph (DBG) construction of the assembler in augmenting short reads with longer reads. The single reads assembly was further refined using the long-read assembler DBG2OLC (Figure 1B), which is an efficient assembler for large genomes, and uses long erroneous reads to build the assembly backbone. Therefore, this precisely assembled genome of *Jatropha* using hybrid methodology forms an accurate reference genome for various downstream applications like MAS, GWAS, re-sequencing programs, and genomic selections aimed for improvement of *Jatropha* for targeted traits. 

In this study, different type of SSR motifs were found, as shown in Table S7, and in total, 18,300 SSRs were identified. This number is less when compared to other Euphorbiaceae members [66,67]. This could be due to a lower mutation rate, the short evolutionary history of the *Jatropha* species and their isolation to certain geographical pockets, and the relative non-exploitation or human intervention of the species. SSRs have been proven as powerful tools in genetic analyses, development of linkage map, QTL mapping, and other genomic studies. The shortlisted SSR markers developed in this study were subjected to genetic diversity, parental polymorphism studies, genotyping, linkage map construction, and the development of QTLs for geminivirus resistance. This represents the first linkage map of *Jatropha* that could provide in the future an indispensable and powerful tool for QTL analysis, gene mapping, candidate gene identification, and marker-assisted breeding for geminivirus resistance. Genomic analyses of these QTL interval regions revealed putative genes such as LRR receptor-like serine/threonine-protein kinase At3g47570, ABC transporter G family member and GDSL esterase/lipase 4-like, which are mainly involved in combating against biotic and abiotic stresses in *Jatropha*. Further, fine mapping and complement testing of these individual genes would provide insights into disease resistance mechanisms.

Now that we are equipped with resources such as the availability of whole-genome sequences of the majority of plants, a comparative genomics and repeat analysis study at the gene level is more feasible. In this comparative genomics study, we observed the clustering of highly conserved proteins of related and distant species with *Jatropha*, which are essential for plant growth and functions. Utilizing such data, we can answer questions by considering the conserved and common features in same or distinct family members. In addition, the data can be used to discriminate specific patterns within subspecies, and to identify the genomic variations or genetic markers that are associated with certain phenotypes or traits. Such information will be very vital for molecular biologists and agriculture researchers to exploit the potentials of the crop under consideration [68]. The comparative genomics study might allow other oil-producing species to leverage unique characteristics that are found in *Jatropha*. As it is a drought-tolerant species, candidate genes related to this trait can be used for other oil producing plants, such as sesamum, castor, and brassica species that are severely affected by drought stress. In addition, bioinformatics and comparative and differential bioinformatics RNA-seq will benefit the identification of candidate genes for biotic and abiotic stresses, such as virus and drought tolerance, respectively.

In this study, we report a de novo assembled *J. curcas* genome to demonstrate the application of genomic information in biodiversity studies, and in finding QTLs for virus resistance. Although *Jatropha* is well known for adaptability, drought tolerant, and multiple uses, its full potential is yet to be explored. There is still lack of understanding of how to grow improved varieties of *Jatropha* with desirable traits under specific growing conditions. Therefore, improvements in *J. curcas* can be possible through the assessment of variations in naturally existing germplasm, selecting relevant genotypes, and gene transfers through inter- and intra-specific hybridization and the use of biotechnological/molecular biology tools to bring change in desired quantitative and qualitative traits. Through this article on de novo genome sequencing, gene identification, confirmation, and associated studies on molecular marker identification, genetic linkage map development, differential gene expression studies, etc., we feel that the genome of *Jatropha curcas* will be better understood, and that this will be useful in developing the crop as a future source of biofuels and biochemical molecules. We also hope that this study and its sequence information would empower researchers and breeders to improve this crop in terms of per unit field productivity.

## Figures and Tables

**Table 1:** Statistics of the hybrid assembly with a combination of MaSurCa, DBG2OLC, and RNA scaffolding. BUSCO: Benchmarking Universal Single-Copy Orthologs.

Number of contigs	2959
Total bases (bp)	265,767,376
Max contig sequence (bp)	1,050,193
Average sequence (bp)	89,817
N50 length (bp)	169,453
GC content genome (%)	33.40
Protein-coding genes (AUGUSTUS 3.2.1)	20,759
Protein-coding genes (BRAKER2)	18,890
Average gene length (Kb)	1.16
GC content of genes (%)	43.85
Gene space-covered BUSCO (%)	84.8
Repeats identified (%)	41.09

## Supplementary Materials

The following are available online at http://www.mdpi.com/2073-4425/10/1/69/s1, **Figure S1**: Sequencing summary of PacBio reads, **Figure S2**: RJC1 amino acid sequence distribution in various KEGG pathways, **Figure S3**: Log2FPKM (Fragments Per Kilobase of exon per Million fragments mapped) values of 620 differentially expressed genes in geminivirus-infected and young leaves from RNA-seq experiments, **Figure S4**: Genetic relationship between 49 *Jatropha* accessions by UPGMA cluster analysis. The Y-axis depicts the two major groups, including Asiatic (*J. curcas* [RJC: 1-24]; *J. integerrima* [RJIP: 1-8]) and Central American (*J. curcas* [RJCA: 1-14]; *J. gaumeri* [RJG: 1-3]) lines, **Table S1**: Statistics of RNA-seq reads of 13 different *Jatropha* tissues at various developmental stages, **Table S2**: Different *Jatropha* species used in the diversity analysis for the characterization and validation of the newly developed SSR markers, **Table S3.1**: Statistics of raw reads of paired-end and mate-pair libraries of RJC1, **Table S3.2**: Statistics of raw reads of PacBio read libraries of RJC1, **Table S4**: Statistics of the PacBio subread length distribution of RJC1, **Table S5.1**: Statistics of high quality Illumina clean reads of RJC1, **Table S5.2**: Statistics of high quality error-free PacBio reads of RJC1, **Table S6**: The table shows the DEGs [up regulated (green) and down regulated (red)] with corresponding FPKM values, fold-change, *p*-value, and pathway name.VI: Virus-infected leaf and YL: Young leaf, **Table S7**: Gene ontology classification of putative genes associated with drought tolerance in the *Jatropha* genome, **Table S8**: Statistics of simple sequence repeat (SSR) markers and designed primers, **Table S9**: The number of markers with corresponding genetic lengths for each linkage group of *Jatropha*, **Table S10**: Summary of the minor QTLs detected in RJC1 with marker intervals, corresponding LODs, and estimated linkage distances of QTLs in Kosambi centimorgans (cM).
