# Supplementary material for: De Novo Sequencing and Hybrid Assembly of the Biofuel Crop Jatropha curcas L.: Identification of Quantitative Trait Loci for Geminivirus Resistance
Source: Genes (Basel). 2019 Jan 21;10(1):69. doi: 10.3390/genes10010069 (PMC6356885; doi:10.3390/genes10010069)

Supplementary Tables

Table S1: Statistics of RNA-seq reads of 13 different *Jatropha* tissues of various developmental stages.

S.No	Tissue	Raw read counts	Clean read count
**1**	Plumule	20,970,012	17,166,382
**2**	Geminivirus infected leaf	9,332,490	7,735,486
**3**	Developing seed 19 DAP	11,522,389	9,268,009
**4**	Developing seed 33 DAP	10,425,440	8,080,963
**5**	Developing seed 40 DAP	9,900,484	8,100,396
**6**	Embryo from germinating seed 24 HAI	28,187,920	21,290,876
**7**	Embryo from germinating seed 36 HAI	33,030,445	23,336,184
**8**	Embryo from germinating seed 48 HAI	37,852,720	27,051,944
**9**	Embryo from germinating seed 72 HAI	35,450,950	24,900,667
**10**	Young shoot	8,890,469	7,442,022
**11**	Male bud	12,511,501	9,158,419
**12**	Radicle	23,722,258	19,418,220
**13**	Young leaf	7,347,484	5,348,169

DAP: days after pollination ; HAI: hours after imbibition

Table S2: *Different Jatropha species used in the diversity analysis for the characterization and validation of the newly developed SSRs markers.*

Region	Species	Accession used	Numbers
**Asia**	*J. curcas*	RJC1- RJC24	24
	J. integerrima	RJIP1- RJIP8	8
**Central America**	*J. curcas*	RJCA1- RJCA14	14
	J. gaumeri	RJG1- RJG3	3

Table S3.1: Statistics of raw reads of paired-end and mate-pair libraries of RJC1.

Library type/Insert size	Paired end (insert size 150bp)	Paired end (insert size 300bp)	Paired end (insert size 550bp)	Mate Pair (1 – 3Kb)
**Number of paired end reads (bp)**	79,178,765	98,605,674	81,412,063	13,252,720
**Number of bases (MB)**	15,835.76	19,721.14	16,282.42	6,626.36
**GC%**	36.2	37.03	38.12	36.08

Table S3.2: Statistics of raw reads for PacBio read libraries of RJC1.

Library type	Pacbio RS II platform
**Number of reads**	1,202,336
**Number of bases (Mb)**	4,722
**GC%**	36.76

Table S4: Statistics of the PacBio subread length distribution of RJC1.

Region	No. of bases	No. of reads	Mean subread Length (bp)
**~ 2kb**	180,307,776	155,176	1,162
**~ 3kb**	231,670,997	95,615	2,423
**~ 4kb**	229,650,548	66,010	3,479
**~ 5kb**	236,849,714	52,822	4,484
**~ 6kb**	246,202,308	44,842	5,490
**~ 7kb**	253,607,557	39,081	6,489
**~ 8kb**	264,339,390	35,289	7,491
**~ 9kb**	272,146,174	32,050	8,491
**~ 10kb**	280,322,805	29,534	9,492
**>10kb**	2,526,829,887	176,558	14,312
**Total**	**4,721,927,156**	**726,977**	**6,495**

Table S5.1: Statistics of high quality Illumina clean reads of RJC1.

Library type	Paired end (insert size 150 bp)	Paired end (insert size 300 bp)	Paired end (insert size 550 bp)	Mate Pair (1 – 3 Kb)
**Number of Paired end reads**	69,537,177	83,485,904	67,847,024	8,301,438
**Number of bases (Mbp)**	13,115.59	15,846.61	12,791.58	2,468.19
**GC%**	35.7	36.6	37.6	35.6

Table S5.2: Statistics of high quality error free PacBio reads of RJC1.

Library type	PacBio RS II platform
**Number of reads**	4,47,402
**Number of bases (Mb)**	1,859
**GC%**	34.73

Table S6: The table shows the DEGs [up regulated (green) and down regulated (red)] with corresponding FPKM values, fold-change, p-value, and pathway name.VI: Virus- infected leaf and YL: Young leaf

S. No.	In-house gene IDs	Gene Names	FPKM		Log2 Fold change	P value	Pathway Name
			VI	YL			
1	g13734	1-deoxy-D-xylulose-5-phosphate synthase 2	5.12913	12.8893	-1.32	0.0436	Metabolic pathways; biosynthesis of secondary metabolites; thiamine metabolism
2	g28639	Ein3-binding protein	33.1462	91.1706	-1.46	0.0162	MAPK signaling pathway; plant hormone signal transduction
3	g27627	Farnesyl diphosphate synthase	16.858	3.86384	2.13	0.0179	Metabolic pathways; biosynthesis of secondary metabolites; terpenoid backbone biosynthesis
4	g29058	ATP synthase subunit alpha, chloroplastic	13.9267	3.86006	1.85	0.0218	Metabolic pathways; photosynthesis; oxidative phosphorylation
5	g30905	Photosystem II protein D1	13.9002	3.56927	1.96	0.0398	Metabolic pathways; photosynthesis
6	g18809		72.9043	18.675	1.96	0.0027	
7	g30628		41.216	11.2958	1.87	0.0154	
8	g30283		56.9478	20.7594	1.46	0.0171	
9	g27170	Ribulose-1,5-bisphosphate carboxylase/oxygenase large subunit	198.11	72.3537	1.45	0.0109	Metabolic pathways; carbon metabolism; carbon fixation in photosynthetic organisms
10	g26610		1408.18	337.866	2.06	0.0272	
11	g30962		16.2622	1.79002	3.18	0.0085	
12	g6957		772.216	268.687	1.52	0.0176	

Table S7: Gene ontology classification of putative genes associated with drought tolerance in the *Jatropha* genome.

Drought related description			JC genes
**Physiological adaption**	Ion and osmotic homeostasis (stomatal movement).		667
	Growth control (root/leaf development).		28
	Detoxification (protection factor, osmolyte production, removal of ROS).		196
**Molecular adaption**	Functional proteins (protection factors, phospholipid metabolism, enzyme for osmolyte biosynthesis, proteases, detoxification signaling, channels and transporters).		431
	**Regulatory proteins**	Gene expression (transcription factors, miRNA, histone modification, chromatin).	632
		Post translation modification (ubiquitin ligases).	107
		Signal transduction (protein kinases, protein phosphates, others).	1,156
		Hormone signaling (ABA biosynthesis, ABA degradation, ABA signaling).	282
		Acid anhydride hydrolases.	36
**Total genes**			**3,535**

Table S8: Statistics of simple sequence repeat (SSR) markers and designed primers.

Type of repeat	Total no. of SSRs predicted	No. of SSR primers designed
**Di nucleotide (p2)**	18,300	12,103
**Tri nucleotide (p3)**	8,574	5,946
**Tetra nucleotide (p4)**	1,557	1,024
**Penta nucleotide (p5)**	257	183
**Hexa nucleotide (p6)**	122	87

Table S9: The number of markers with corresponding genetic lengths for each linkage group of *Jatropha*

LG	No. of Markers	Length (cM)	cM/Marker
**1**	40	885	22
**2**	24	488.2	20
**3**	25	369.7	15
**4**	20	326.8	16

**5**	7	87.9	12
**6**	6	72.8	12
**7**	26	445.1	17
**8**	24	310.2	13
**9**	10	84.4	8
**10**	16	228.8	14
**11**	9	132.3	14
**Total**	**207**	**3431.2**	

Table S10: Summary of the minor QTLs detected in RJC1 with marker intervals, corresponding LODs and estimated linkage distances of QTLs in Kosambi centimorgans (cM).

S.No	QTL	Marker Interval	LOD	cM distance	Major putative genes
**1**	qJMV-3	RJM613 - RJM1472	4.5	37.0	AP2-like ethylene-responsive transcription factor ANT, cytochrome P450 93A3-like
**2**	qJMV-4	RJM236 - RJM1157	3.1	30.6	Retrovirus-related Pol polyprotein from transposon TNT 1-94;<br>Serine/threonine-protein kinase ATR isoform X2;<br>Taxadiene 5-alpha hydroxylase
**3**	qJMV-10	RJM1836 - RJM2234	3.3	43.6	Cytochrome P450 93A3-like;<br>Probable LRR receptor-like serine/threonine-protein kinase At3g47570;<br>TMV resistance protein N-like isoform X1;<br>Serine/threonine-protein kinase ATR isoform X2;<br>Taxadiene 5-alpha hydroxylase

Supplementary Figures

Figure S1: Sequencing summary of PacBio reads.

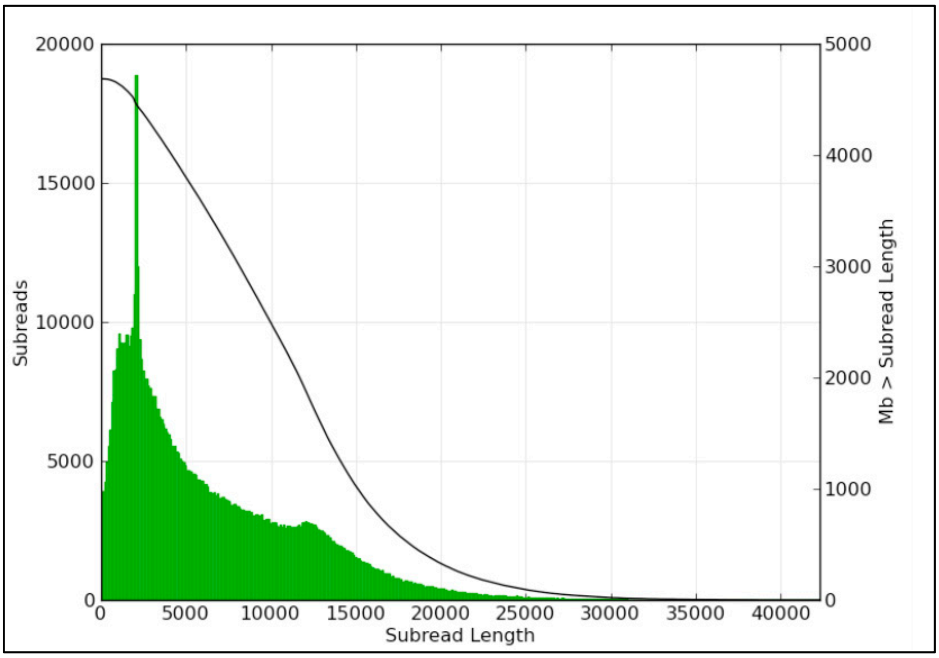

Figure S2: RJC1 amino acid sequence distribution in various KEGG pathways.

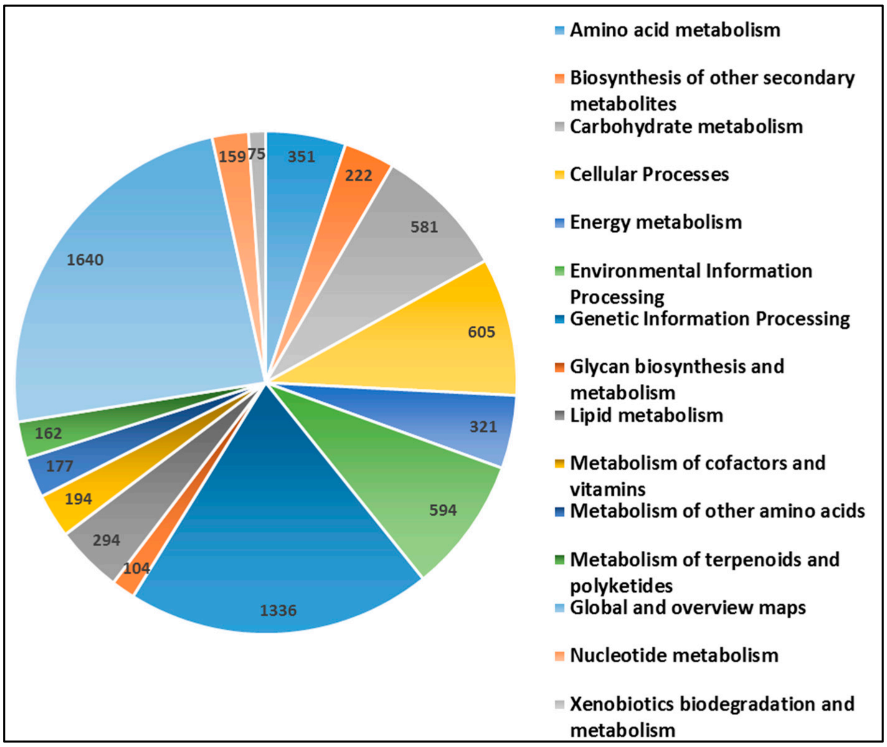

Figure S3: Log2FPKM (Fragments Per Kilobase of exon per Million fragments mapped) values of 620 differentially expressed genes in geminivirus-infected and young leaves from RNA-seq experiments.

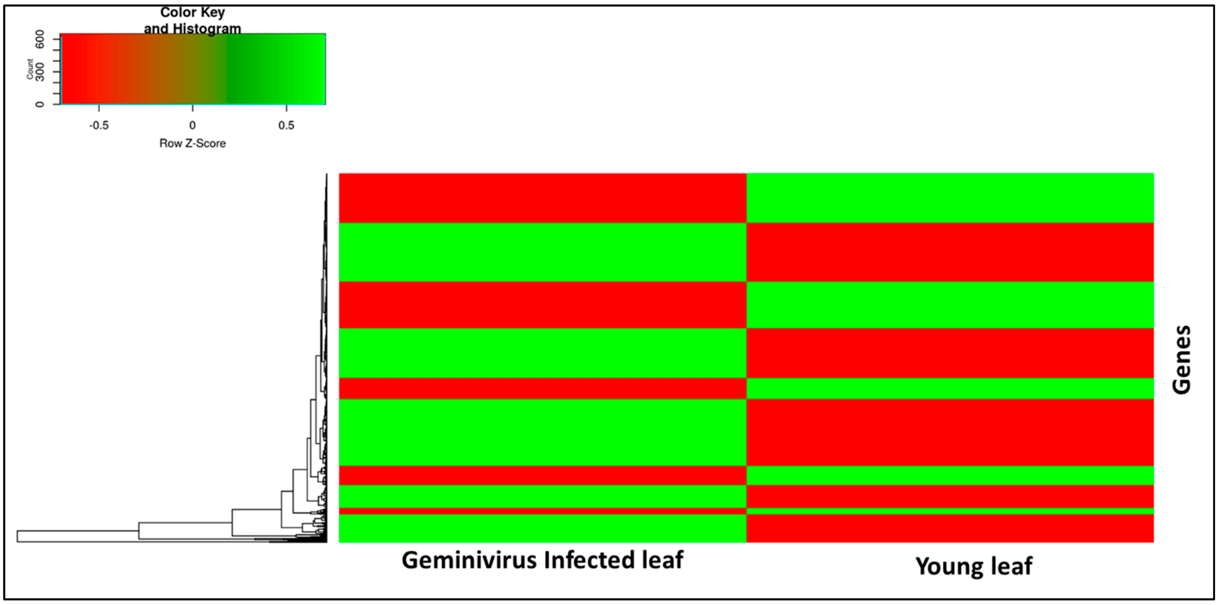

Figure S4: Genetic relationship between 49 *Jatropha* accessions by UPGMA cluster analysis. The Y-axis depicts the two major groups including Asiatic (*J. curcas* [RJC: 1-24]; *J. integerrima* [RJIP: 1-8]) and Central American (*J. curcas* [RJCA: 1-14]; *J. gaumeri* [RJG: 1-3]) lines.

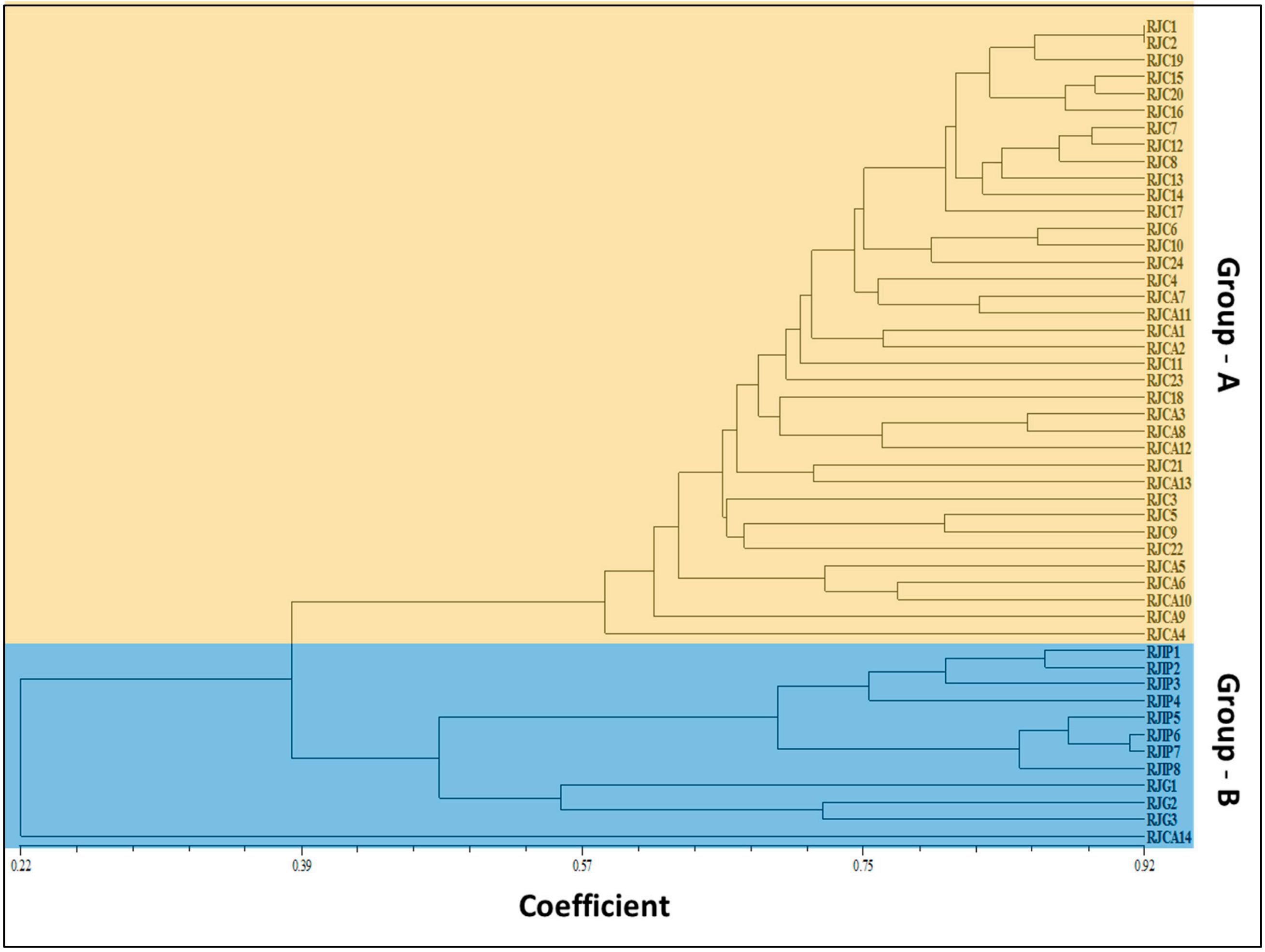

Supplement: Supplementary file 1 [file genes-10-00069-s001.pdf]
